# A three-dimensional algorithm for precise measurement of human auricle parameters

**DOI:** 10.1038/s41598-024-61351-5

**Published:** 2024-05-10

**Authors:** Yangyang Lin, Johannes G. G. Dobbe, Nadia Lachkar, Elsa M. Ronde, Theo H. Smit, Corstiaan C. Breugem, Geert J. Streekstra

**Affiliations:** 1 Department of Plastic, Reconstructive and Hand Surgery, Amsterdam UMC Location University of Amsterdam, Amsterdam, The Netherlands; 2 Amsterdam Reproduction and Development Research Institute, Amsterdam, The Netherlands; 3 Department of Biomedical Engineering and Physics, Amsterdam UMC Location University of Amsterdam, Amsterdam, The Netherlands; 4 Amsterdam Movement Sciences, Musculoskeletal Health—Restoration and Development, Amsterdam, The Netherlands; 5 Department of Orthopedic Surgery and Sports Medicine, Amsterdam Movement Sciences, Amsterdam UMC, University of Amsterdam, Amsterdam, The Netherlands; 6 Department of Medical Biology, Amsterdam UMC Location AMC, Amsterdam, The Netherlands; 7 Department of Gynaecology and Amsterdam Reproduction and Development, Amsterdam UMC Location VUMC, Amsterdam, The Netherlands

**Keywords:** Auricle parameters, CT-imaging, 3D Image analysis, Objective measurement, Precision, Anatomy, Medical research, Preclinical research

## Abstract

Measurement of auricle parameters for planning and post-operative evaluation presents substantial challenges due to the complex 3D structure of the human auricle. Traditional measurement methods rely on manual techniques, resulting in limited precision. This study introduces a novel automated surface-based three-dimensional measurement method for quantifying human auricle parameters. The method was applied to virtual auricles reconstructed from Computed Tomography (CT) scans of a cadaver head and subsequent measurement of important clinically relevant aesthetical auricular parameters (length, width, protrusion, position, auriculocephalic angle, and inclination angle). Reference measurements were done manually (using a caliper and using a 3D landmarking method) and measurement precision was compared to the automated method. The CT scans were performed using both a contemporary high-end and a low-end CT scanner. Scans were conducted at a standard scanning dose, and at half the dose. The automatic method demonstrated significantly higher precision in measuring auricle parameters compared to manual methods. Compared to traditional manual measurements, precision improved for auricle length (9×), width (5×), protrusion (5×), Auriculocephalic Angle (5–54×) and posteroanterior position (23×). Concerning parameters without comparison with a manual method, the precision level of supero-inferior position was 0.489 mm; and the precisions of the inclination angle measurements were 1.365 mm and 0.237 mm for the two automated methods investigated. Improved precision of measuring auricle parameters was associated with using the high-end scanner. A higher dose was only associated with a higher precision for the left auricle length. The findings of this study emphasize the advantage of automated surface-based auricle measurements, showcasing improved precision compared to traditional methods. This novel algorithm has the potential to enhance auricle reconstruction and other applications in plastic surgery, offering a promising avenue for future research and clinical application.

## Introduction

The auricle exhibits highly complex 3D morphological characteristics. Quantitative research on auricular morphology provides an essential reference for the diagnosis and treatment of various congenital ear deformities^1–3^, including protruding ears^3,4^, microtia/anotia^5^, and constricted ears^6^. Precise assessment of the protrusion distance and the auriculocephalic angle is crucial for the diagnosis of protruding ears and serves as the foundation for insurance companies to reimburse this surgery in certain European countries^4^. These parameters, along with aesthetic parameters like auricle length and width, are commonly used for post-operative efficacy evaluations in otoplasty and auricle reconstruction^5^. The inclination angle is also recommended by the UK microtia standard of care for post-operative reconstructive assessment^7^. Assessing similarity in these aesthetic parameters between the reconstructed and normal auricle is vital for pre-operative planning and post-operative evaluation^8,9^.

An example of pathology where quantitative measurement of numerical aesthetic variables is of particular importance is protruding ears. Protruding ears occur in about 5% of the general population. In this patient group otoplasty is a commonly performed surgical procedure. The precise indication for surgery is often defined by measuring protrusion of the ear. However, there is also no standardized method to evaluate the outcome of surgery in an objective manner.

A variety of quantitative auricle parameters have been used to objectify reconstruction outcome based on landmarking^9^. Frequently used landmarks include the superaurale and subaurale for measuring length, the preaurale and postaurale for measuring width and the most prominent point for measuring protrusion. These landmarks are sometimes used in combination with facial landmarks, such as the outer canthus or alare, to further reflect auricle-head relationships, such as bilateral position^9^. In most auricle reconstruction research these parameters are measured manually by rulers, protractors or 2D images, even though it has been shown that manual approaches suffer from substantial observer variability^10–13^. With the aid of a more advanced measurement method such as 3D photographing, a few studies claim more precise results^6,14–19^. Nevertheless, these studies still rely on manually placed landmarks. The variability of the manual measurement approach, influenced by intra or inter-observer variability, was not fully investigated in these studies.

Compared to landmark-based methods, a 3D surface-based approach is potentially more precise because it is based on many points instead of a limited number of landmarks as has been shown in a different application for measurement of facial asymmetry^20^. Once the 3D auricular surface is available, it is not only possible to assess the auricle morphology itself^21,22^ but it also allows quantifying the auriculocephalic (AC) angle between the auricle and the facial surface^23^. An automated approach for quantifying the auricular biometrics could provide a solution in objectifying planning and evaluation of surgical and non-surgical treatment of a variety of ear deformities.

Therefore, the aim of this study is to present and evaluate an automated, objective 3D approach to quantify geometrical (shape) and topological (position) auricle parameters from segmentations of CT-images, based on automatically selecting surfaces and extracting surface-based auricle parameters. We hypothesize that these automatic measurements have a higher precision of measurement than traditional manual methods. To test this hypothesis, we compare the precision of the novel objective 3D approach with traditional manual measurement methods that use physical calipers and 3D landmarking software. We also hypothesize that radiation dose and CT-scanner type may influence image quality and consequently the precision of automated auricle parameter measurement. Therefore, two radiation doses and two different CT-scanners are used to investigate their influence on measurement precision.

## Methods

This study used three techniques to measure auricle parameters: (1) manually, using a caliper, (2) manually, with 3D software using a landmarking function, and (3) using a novel approach that was implemented in custom-made software and enabled measuring auricle parameters in an automated fashion. The latter two techniques are based on measurements on virtual models derived from CT image data of a cadaver head. All three approaches provided commonly used auricle parameters according to clinical practice at the Amsterdam University Medical Centers and are also used in auricle-reconstruction literature, i.e., auricle length, width, protrusion distance/angle, inclination angle and position. Table 1 gives an overview of the auricle parameters that were measured in this study using each of the three methods.

**Table 1:** Auricle parameters as measured in this study using the three measurement approaches (caliper, landmarking using virtual head models and 3D software, the novel surface-based and automated approach using virtual head models).

Auricle parameter	Caliper	Landmarking	Quick reference of Manual approach	Automatic	Quick reference of Automatic approach
Auriculocephalic angle vs mastoid skin		☑	The angle between the back side of the auricle and the mastoid at the point where a horizontal line through the otobasion superius meets the helix	☑	Angle between the normal vectors of the planes fit through the auricle surface and the mastoid surface
Auriculocephalic angle vs midsagittal plane				☑	Angle between the normal vectors of the plane fit through the auricle surface and the mid-sagittal plane
Protrusion distance	☑	☑	Perpendicular distance from the most lateral (prominent) point of the auricle to the mastoid	☑	Distance between most-lateral auricle point and plane fit through the mastoid surface
Length	☑	☑	Distance from the Superaurale to the Subaurale	☑	(1) Longest distance between two points, (2) Longest axis after PCA
Width	☑	☑	Distance from the Preaurale to the Postaurale	☑	(1) Distance perpendicular to length line 1) (2) Second longest axis after PCA
Inclination angle				☑	Angle between the longest axis after PCA and the vertical axis of the coordinate system
Posterior-Anterior Position	☑	☑	Bilateral difference of distance from the outer canthus to the otobasion superius (OS)	☑	Distance (along z axis, see Fig. 1c) between center positions of the left and right auricle, projected in the mid-sagittal plane
Superior-inferior Position				☑	Distance (along y axis, see Fig. 1c) between center positions of the left and right auricle, projected in the mid-sagittal plane

*PCA* principal component analysis.

The two manual approaches were repeated by three researchers from the department of plastic, reconstructive and hand surgery at different time points. By pooling the results of these observers, we include inter- and intra-rater variability in the manually obtained data. Variability in the automated approach is mainly due to image noise, which depends on the quality of the scanner and on the scan protocol. To include this variability in the measurements based on virtual models, multiple CT scans were made of the cadaver head in different positions within the CT scanner, with different CT devices and using different radiation doses.

### Virtual head reconstruction

#### Cadaver specimen and CT image acquisition

A cadaver head was obtained through the body donation program from the Department of Medical Biology, Section Clinical Anatomy and Embryology, of the Amsterdam UMC at the location Academic Medical Center in The Netherlands. The body from which the sample was taken was donated in accordance with Dutch legislation and the regulations of the medical ethical committee of the Amsterdam UMC at the location Academic Medical Center. Both auricles were intact, but the right auricle was covered with ice. We thawed both auricles under tap water at room temperature to restore the shape of the auricles, and re-frost it using liquid nitrogen to fix the shape of the auricles. No clear craniofacial abnormalities and deficiencies were observed.

Twenty standard CT sinus scans were made of the frozen cadaver head including both auricles, eyebrows, nose, and mouth. Ten of the scans were made on a high-end Siemens Somatom Force CT scanner (Siemens Healthineers, Erlangen, Germany, voxel size 0.46 × 0.46 × 0.45 mm, 120 kVp). To enable calculation of variability in the measurement parameters, the scans were repeated for each scanning condition. Five scans were made on a standard dose (160 mAs), and five on half the dose (80 mAs). Another ten scans were made with the same scanning parameters on a low-end Siemens Sensation 64 CT-scanner (Siemens Healthineers, Erlangen, Germany). The cadaver head is repositioned after each CT scan. Segmentation of the heads in these scans provided a total of twenty virtual head models for further analysis.

#### Segmentation procedure

**Figure 1:**
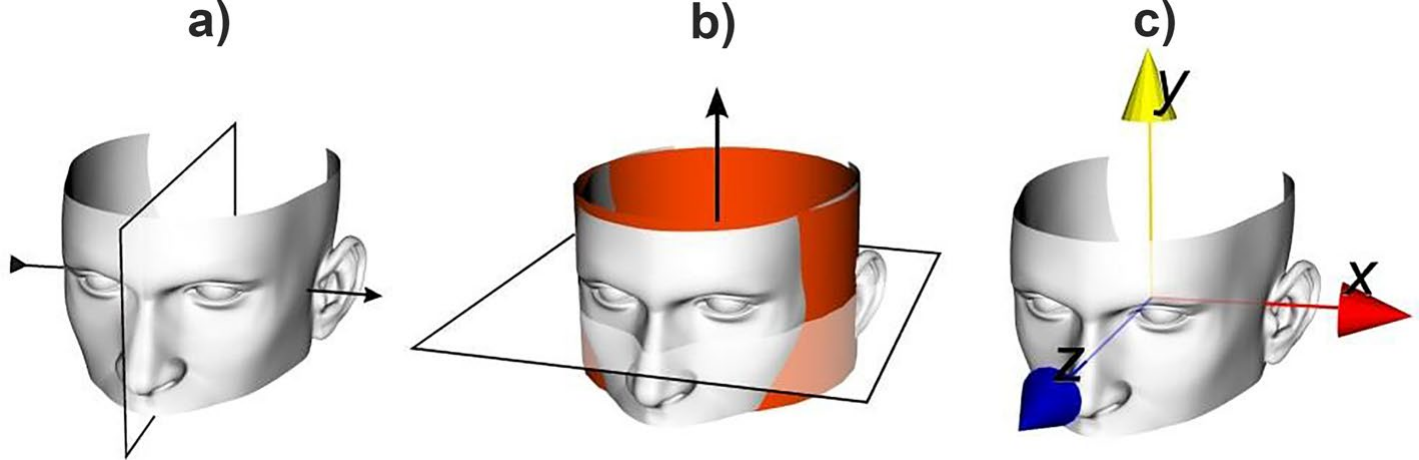
Definition of an anatomical coordinate system of the head. (**a**) The + x-axis is perpendicular to the plane of symmetry of the head, i.e., the sagittal plane, and points in the direction if the left ear. (**b**) The y-axis is found by cylinder fitting of the head surface. The cylinder axis in the superior-inferior Position direction serves as + y-axis. (**c**) The + z-axis is perpendicular to the + x- and + y-axes.

For evaluation of the auricle parameters using virtual head models, the head was segmented using custom-made software^24^ in a two-step approach. In the first step, the bony skull tissue was segmented, followed by a closing algorithm^25^ to fill existing bony cavities (bony orbit, sinus cavities, ear canal, etc.) at a higher threshold (> 500 HU). In the second step the soft tissue was added using the same region growing approach at a lower threshold (> − 500 HU). By using the closing algorithm only in the first step, we closed cavities but avoided closing irregularities at the auricle surface. The image voxels segmented this way initialized a Laplacian level-set segmentation growth algorithm^26^ which advances pixel dispersion towards the outline of the soft tissue towards a high intensity gradient. The gray-level image was filtered using Gaussian smoothing (SD = 1 mm) prior to starting this iterative procedure (set to 20 iterations). Finally, the marching cubes algorithm^27^ was used to extract a polygon mesh at the zero-level of the level-set image. The polygon meshes (Fig. 1) represented the 3D virtual surface-mesh models of the heads and were used for auricle parameter measurement, either manually or in an automated fashion.

### Measuring auricle parameters

**Figure 2:**
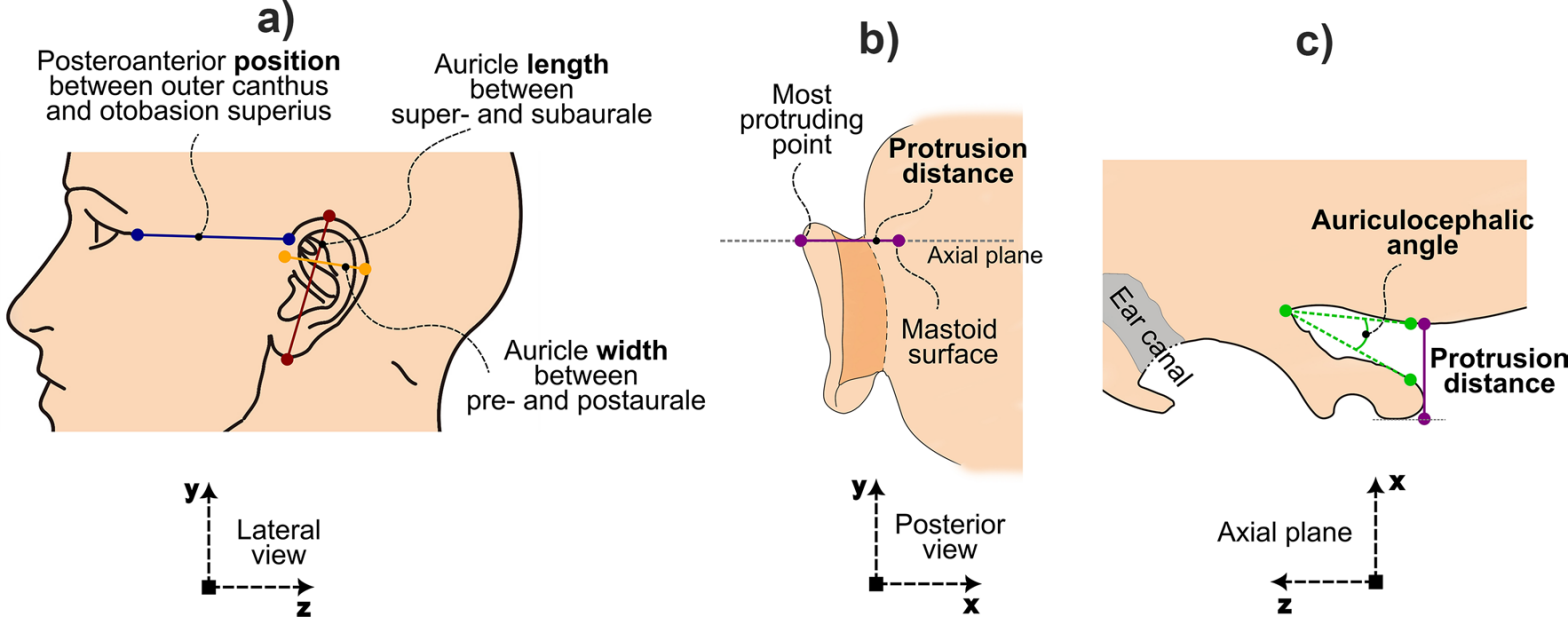
Manual (caliper and 3D landmarking) measurements. (**a**) Lateral view of the head, showing length (red), width (yellow), distance OS (blue). Noted that position measured manually is presented as difference of bilateral distance OS. (**b**) Posterior view of the head, showing protrusion distance (purple) and the place to Axial plane (gray). (**c**) Axial plane view of the head, showing auriculocephalic angle (green) and protrusion distance (purple). The coordinating systems are in line with those in Fig. 1.

Auricle parameters were selected for the manual measurements using caliper, manual measurements using landmarking software and using the automated approach. Not all parameters can be easily determined using all three approaches. For example, the auriculocephalic angle and inclination angle are quite challenging to measure using a caliper. Table 1 and Fig. 2 show which parameters were measured using each of the three approaches.

#### Manual measurement using a caliper

Three researchers (YYL, EMR, and NS) from the Plastic, Reconstructive and Hand Surgery Department at Amsterdam University Medical Centers, location Academic Medical Center, independently measured the two auricles on the cadaver head using a caliper (IRONSIDE®). The manual measurements using a caliper were conducted in two rounds, with an interval of at least one week between each round. In each round of measurement, each researcher measured each auricle five times. This approach yields inter-observer variability and intra-observer variability for every auricle parameter in Table 1 (see also Fig. 2a,b).

#### Manual measurements using virtual heads

The same three researchers independently measured auricle parameters for both auricles using the 3D landmarking approach in MIMICs software (Materialise, Leuven, Belgium). In this approach the user is enabled to pinpoint locations on the 3D surface of the virtual head using a mouse click according to the definitions of Table 1, to measure distances or angles. The measurement methods of protrusion distance, length, width and positions are the same with the caliper measurements in 2.2.1 (Fig. 2a,b), while the auriculocephalic angle (Fig. 2c) was additionally measured on a virtual head using the 3D landmarking approach. The manual measurements using 3D landmarking software were also conducted in two rounds, with an interval of at least one week between each round. In each round, all researchers measured the auricle parameters five times. This approach included inter- and intra-observer variability for every landmarking parameter listed in Table 1. To minimize variability caused by noise in the CT-images, we selected the model that was obtained using the best CT scan made using the high-end Siemens Somatom Force CT scanner at the highest dose (160 mAs).

#### Automatic measurements using virtual heads

The twenty virtual heads reconstructed from the 20 CT-scans of the cadaver head were evaluated using custom made software by one researcher to measure geometric parameters (Table 1) of 40 auricle representations in the segmentations of the 20 CT images. The analysis algorithm is as follows (specific details of the analysis algorithm are described below): for each head, a 3D coordinate system was determined (Fig. 1c) based on the mid-sagittal plane, of which the plane normal vector defined the + x-axis from the right auricle to the left auricle. The y-axis was found by fitting a cylinder through the mesh points of the head and using the Caudocranial centerline as + y-axis. The + z-axis was perpendicular to the + x- and + y-axes using the right-hand rule. The centroid of our head model is projected in the mid-sagittal plane and defines the origin of our coordinate system. The coordinate system is used to quantify the inclination angle and auricle-positioning parameters.

**Figure 3:**
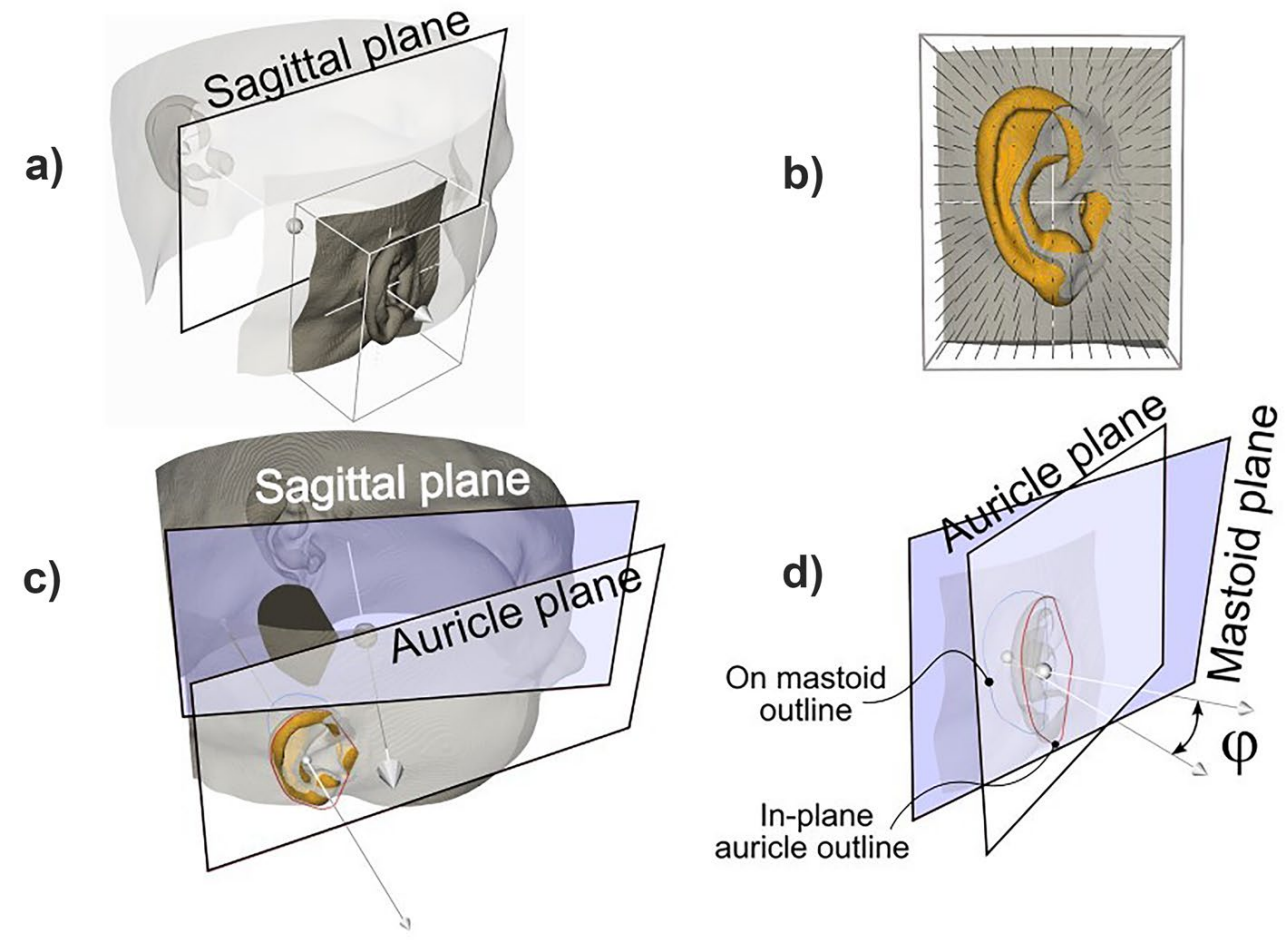
(**a**) A 3D box parallel to the sagittal plane selects a region of the head to be evaluated. (**b**) A dense grid of lines intersect the selection in (**a**) at one or more locations. The most lateral points of intersection are selected, if at least two intersections are found, to exclude the surface around and behind the auricle. A triangular mesh connected the selected points and completed selection of the auricle surface (orange). (**c**) A plane is fit through the selected mesh points which serves as the auricle plane, and the auricle surface is projected into this plane. The hull (red outline) is projected into the sagittal plane (black) and then projected onto the mastoid surface of the head model (blue outline). The angle between the sagittal plane and the auricle plane defines the auriculocephalic angle. (**d**) A plane is fit through the blue outline on the mastoid surface and provides the mastoid plane. The angle between the normal vectors of the auricle plane and the mastoid plane provides the auriculocephalic angle.

Next, an auricle selection algorithm was used to extract the auricle surface from the virtual head. Least-squares fitting was used to find the plane through this auricle surface: the auricle plane. To also find the plane describing the mastoid surface, while excluding the inner ear canal from the mastoid surface, the following procedure was used. The auricle surface was projected into the auricle plane, and the hull, i.e., the outline of the projected auricle, was projected in the mid-sagittal plane, and then back onto the mastoid surface of the head (Fig. 3c). Least-squares fitting was again used to find the plane through this mastoid outline: the mastoid plane.

##### Midsagittal plane (Fig. 1a***)***

The midsagittal plane was found by a minimization algorithm that iteratively chooses a plane in which the virtual head model is mirrored while evaluating a cost function. The average nearest-neighbor distance between points of the original head model and the mirrored model served as cost function in our implementation. To this end, the Nelder-Mead downhill simplex minimization algorithm^28,29^ was implemented with a three-parameter search space: longitude and latitude angles defining the plane's normal vector, and hence the plane orientation, and the distance from the CT origin to the plane, as measured in the direction of the plane's normal vector. For each iteration, the average and standard deviation of the distances were determined and outliers with a distance larger than the mean + 0.5 SD, were excluded to calculate the cost function. This approach allows some degree of asymmetry, e.g., a missing auricle, while still being able to find a feasible plane of symmetry. The user initialized the procedure by interactively placing a first guess of the mid-sagittal plane.

##### Cylinder fitting (Fig. 1b)

The y-axis was found by fitting a cylinder to the head model and using the cylinder axis in the superior-inferior (S-I) direction as + y-axis. To this end, another minimization algorithm was implemented based on the Nelder-Mead downhill simplex minimization algorithm^28,29^ in a similar way as for the aforementioned midsagittal plane. In this case with a six-parameter search space: longitude and latitude angle to define the cylinder orientation, (x, y, z) that define the origin of the coordinate system, and the cylinder radius. For each iteration a cylinder mesh was created based on these six-cylinder parameters, containing 250 points across the cylinder surface. The average nearest-neighbor distance between points of the head model and the cylinder again served as cost function and outliers were excluded from the virtual head model in the same way as described above. The user initialized the procedure by interactively placing a first guess of the cylinder’s centerline, while the average distance to this line was used as initial guess for the cylinder radius.

##### Auricle surface selection

A 3D box with the surface normal of the lateral and medial faces parallel with the x-axis of the local coordinate system (see Fig. 3a), and the normal vectors of the cranial and caudal faces parallel with the y-axis, was manually resized and translated to enclose each auricle and to avoid including unintended regions, such as the nose. Dense grids (spacing 0.3 mm) of corresponding points in the lateral and medial box face were connected and defined a grid of parallel lines, each intersecting the virtual head model (see Fig. 3b) at one or more locations. Only the most lateral points of intersection between the grid lines and the virtual head model were selected, under the condition that at least two intersections with each projection line were found. This ensures that only the auricle surface is selected since in other regions only one intersection point exists. A triangular mesh connected the lateral auricle points and completed selection of the auricle surface.

##### Auriculocephalic angles

The auriculocephalic angles were quantified and evaluated in two ways: (1) by the angle between the normal vectors of the auricle plane and the mid-sagittal plane (Fig. 3c), and (2) as the angle between the normal vectors of the auricle plane and the mastoid plane, (Fig. 3d). We hypothesize that the latter is affected by the shape of the head as well, while this is not the case for the former approach, which therefore is expected to reflect a more precise way to quantify the protrusion angle.

##### Protrusion distance

The protrusion distance was determined by the largest distance from the mastoid plane to any point in the selected auricle surface, measured perpendicular to the mastoid plane.

##### Auricle length and width

The points of the auricle surface were used to determine the length and width of an auricle in two ways, to be able to investigate if one method performed better than the other with respect to the variability in assessing these parameter variants. For these approaches the points across the auricle surface were projected into the auricle plane. In the first approach the largest distance between any two of these in-plane points defined a line L and the length of this line served as length parameter. All in-plane points were then projected onto an in-plane line perpendicular to L. The distance between the far-most points after this projection, defined the width of the auricle, and provided the in-plane line W, perpendicular to line L, running through the point furthest from line L. In the second approach, we employed a variant of Principal Component Analysis (PCA) to calculate the inertia tensor from the in-plane points of the auricle. This tensor enabled calculating the two eigenvectors and eigenvalues. The eigenvector with the smallest inertia defined the direction of the length axis, and the other eigenvector defined the direction of the width axis. The length and width of the rectangle in line with these axes, bounding the in-plane auricle points represented the auricle length and width.

##### Auricle position

The center of the above-mentioned box represented the center of an auricle. By projecting these center points for the left and right auricle into the sagittal plane we were able to find the posterior-anterior auricle translation (measured along the z axis) and the superior-inferior auricle translation (measured along the y-axis). The posterior-anterior auricle translation is considered to be the automated analogy of the manually measured bilateral difference of the distance from the outer cantus to the otobasion superius (Table 1). For the superior-inferior auricle translation, there is no evident manual caliper or landmarking method that reflexes this auricle position parameter.

##### Inclination angle

The axis determined by auricle length was projected in the mid-sagittal plane, and the angle between this projected axis and the y-axis of the coordinate system provided the auricle inclination angle. Thus, there are two ways of measuring the inclination angle: one based on the auricle length found by the PCA, and the other one found by the diameter.

### Statistics

The data was analyzed using R studio (RStudio 2022.02.3 + 492 “Prairie Trillium” Release). Levene’s test was employed to compare variabilities of the same parameter across different measurement methods. In this study we focus on measurement precision (i.e. the standard deviation (SD) among multiple repetitive measurements), but we also use Anova and post hoc tests to evaluate if differences in the mean values of the different measurement approaches exist and to what extent they deviate, although we will not be able to conclude which approach is the best since gold standard values of the measurement parameters are missing. Multivariate linear regression analysis was used to determine the contribution of different automatic measurement methods (PCA- vs. diameter-based approaches), CT scanner types (Siemens Somatom Force vs. Siemens Sensation) and radiation dose (high vs. low) to the variation of the measurement.

## Results

### Precision: automatic measurements vs. manual measurements

**Figure 4:**
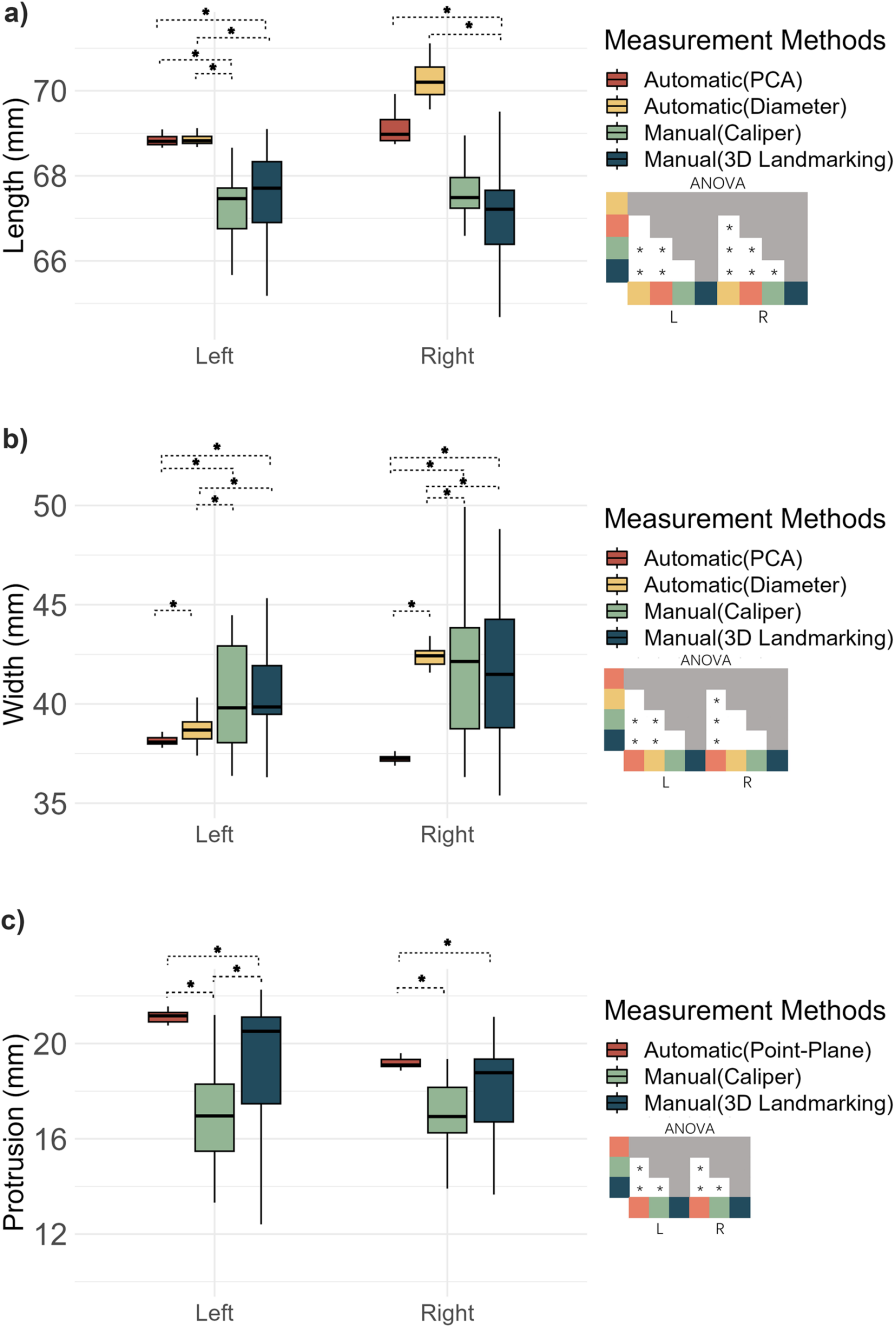

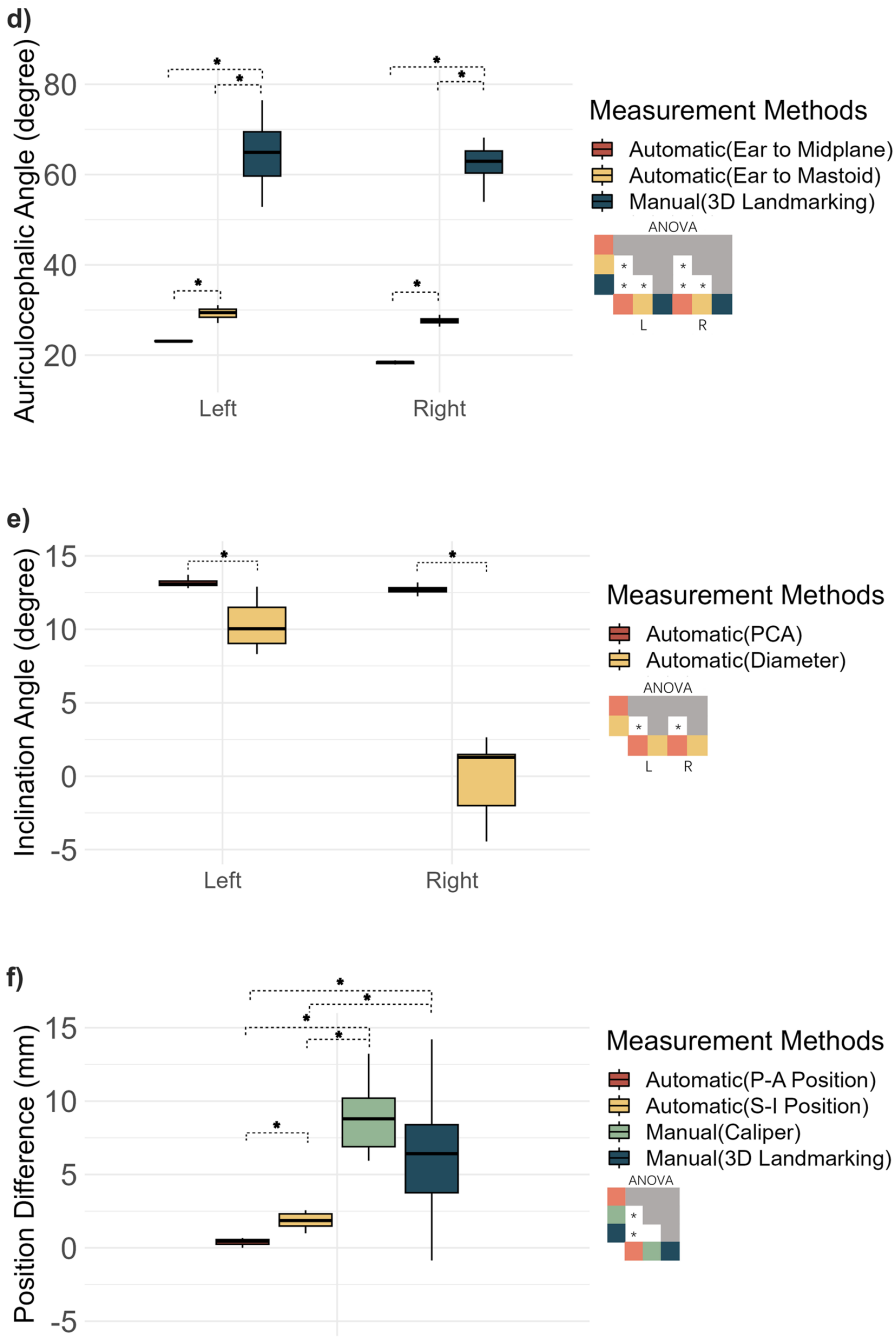
Precision discrepancy between automatic and manual measurement methods across various parameters. This figure comprises six plots. Each plot represents a distinct auricle parameter: length, width, protrusion, auriculocephalic angle, inclination angle, and position difference. The plots (**a**)–(**e**) feature left and right auricle on the x axis, with means of repetitive measurements of each auricle parameter on the y-axis. Plot (**f**) depicts the difference of means of bilateral positions on the y-axis, hence featuring only one x-axis label. The significance differences atop each box indicate the statistical disparity in standard deviations between different measurement methods. Mean differences under different measurement methods are showcased in the ANOVA heat maps on the right side of each of the parameter. Gray boxes indicate that there were no need to compare the difference of means, as these are already compared in the white boxes. In scatter plot of position (**e**), P–A means posterior–anterior and S–I means superior–inferior. The mean difference of S-I was not show because no manual approach was available to be compared.

The measurement precision (SD) was significantly higher for almost all parameters obtained using the automated approach (Fig. 4), except for the length of the right ear (PCA vs. manual caliper: p = 0.09, Diameter vs. manual caliper: p = 0.14). Interestingly, the automatic measurement results of the right auricle length showed a larger variability than that of the left auricle.

Compared to the diameter-based approach, the PCA-based approach showed a significantly higher precision when measuring auricle width, inclination angle, and left auricle length. The PCA-based approach was also associated with a decreased standard deviation in the measurements of the inclination angle and auricle width compared with the diameter-based approach. One exception, however, is that the PCA-based approach increased the standard deviation when measuring the left length (estimate = 0.10 mm, p = 0.01). In addition, the auriculocephalic angle measured with respect to the midsagittal plane was significantly more precise than the auriculocephalic angle with respect to the mastoid plane. In the bilateral positional difference, the posterior-anterior position was more precise than the superior-inferior position. No difference in precision could be confirmed between manual measurements using calipers and landmarking software for these parameters (Fig. 4).

### Precision: effect of scanner type and dose

Precision varied between measurements based on images acquired with the Force and Sensation scanners. A higher precision was associated with using the Force scanner. The relation of this more advanced high-end CT-scanner with higher precision can be confirmed statistically only for length of both auricles, and right inclination angle in the multivariate linear regression analysis. A higher precision was only statistically associated with a higher dose for the left auricle length, where a lower dose resulted in a more variable value (estimate = 0.27 mm p < 0.01).

### Mean value: differences between measurement methods

Significant differences were observed for the means of auricle parameters when measured between manual and automated measurements. One exception was the right auricle width. When comparing the mean value of auricle parameters obtained using the automatic PCA-based approach and the diameter-based approaches, all parameters were significantly different, except for measuring the length and width of the left ear. When comparing manual measurement approaches, i.e., using a caliper compared to the 3D landmarking function, it was found that the mean values were all different except for measuring the left auricle length and bilateral width, and the position difference between Caliper and Landmarking. We found many statistically significant differences in the average values of auricle parameters among the different methods. To put those differences in perspective, we also report the relative differences (percentage) in the average values compared to the commonly used manual caliper measurements (Table 2).

**Table 2:** Error percentage of different parameters measured by 3D landmarking and automatic approaches compared to the Manual Caliper.

	Left			Right		
	3D Landmarking	PCA	Diameter	3D Landmarking	PCA	Diameter
Length	1%	2%	2%	1%	2%	4%
Width	0%	5%	4%	2%	12%	1%
	3D Landmarking	Point-plane		3D Landmarking	Point-plane	
Protrusion	14%	24%		6%	13%	
		|Left–Right|				
	3D Landmarking	Posterior–anterior positon				
Position	22%	95%				

This table displays the error percentages for various auricle measurements, comparing results obtained through Manual (3D Landmarking) and Automatic methods with Manual (Caliper). The error percentages are presented separately for the left and right auricles, highlighting variations in measurement accuracy across different parameters and measurement techniques. Note that the Position measurement is calculated as difference of bilateral ears.

## Discussion

Our results demonstrate that the automated surface-based approach generally outperforms both manual measurement types (Caliper and 3D landmarking) in terms of precision, when measuring the aesthetic auricular parameters. This finding is consistent with our hypothesis that automated measurements would offer a higher precision compared to traditional methods. We also found that CT scanner type and dose had little effect on measurement precision. Moreover, the proposed automatic approach added the capability of measuring the inclination angle, the auriculocephalic angle, and the auricle position, which cannot easily be obtained using conventional manual approaches.

The variation in automated measurements for the right auricle appeared to be larger than for the left auricle. This difference in variability may be attributed to the different storage conditions since the left auricle was frozen in dry air, while the right auricle was slightly distorted in bulky ice. It was therefore re-frozen by pouring liquid nitrogen over it. When diving deeper into the higher variability we found that the auricle parameters changed following a trend during the time in which the repeated scans were made. This suggests that the auricle thawed over time, causing a slight change of shape, which may explain the higher variability for the right auricle.

Differences in precision for the PCA and Diameter-based approaches may be explained by the resolution of the grid of points used for projection onto the auricle (see section "Automatic measurements using virtual heads"). The diameter-based approach can be expected to be more sensitive for a discretization error up to the grid spacing (0.3 mm) than the PCA approach, where discretization errors are averaged out by the relatively high number of points used for performing the PCA analysis. The larger effect of the discretization error is also evident from the auricle inclination parameter, which showed a small variation for the PCA approach and a large variation for the diameter-based approach, which is again determined from two grid points (see Fig. 4e). One grid point displacement due the discretization error can have a relatively large effect on the resulting inclination angle. Increasing the grid resolution may reduce this discretization error. We recommend using the PCA-based approach to measure auricle length, width and inclination in future studies.

It was found that the auriculocephalic angle measured with respect to the midsagittal plane was more precise compared to using the mastoid plane as reference. Apparently, the mastoid plane adds more variability to the measurement compared to using the midsagittal plane, which means that finding the midsagittal plane is more precise than finding the mastoid plane. This may be explained by the fact that the midsagittal plane is determined from the many points of the mesh representing the virtual head surface, while the mastoid surface is much smaller containing less than 1% of those points. It is therefore likely that finding the midsagittal plane is more precise. The definition of measuring the auriculocephalic angle using the manual landmarking approach is slightly different (see Fig. 2) compared to the automated approach (Fig. 3). As a result, the average auriculocephalic angle was quite different for these approaches. However, the automated approach was much more precise in measuring the auriculocephalic angle than the conventional approach (Fig. 4d).

In this paper we reported the precision of measuring auricle parameters, which in general was better using our novel approach compared to the conventional manual approaches. Available literature on auricular morphology is often unclear in the reliability of the measurement approach that has been used. Moreover, a standard way of reporting measurement precision is lacking, which renders comparison with scarce literature difficult. Chen et al. reported the relative error magnitude as measure of precision in caliper and 3D landmarking measurements for auricle length (1.0%) and width (1.7%). These values translate to a variability measure of pprox.. 0.7 mm for auricle length and 0.5 mm for width, which seem similar to the results of our manual measurements (see Fig. 4a,b). Our automated approach on the other hand was much more precise and can be of value for future auricle morphology studies.

A unique aspect of this study is the validation of our automated method with data from repeated CT scans. We expected that high-end scanner type and higher dose would increase measurement precision. However, for most of the parameters the scanner type and dose were not correlated to measurement precision, which was superior to those of the manual method in all cases. This further means that our method is robust and can be used in both low and high end scanners and with relatively low dose levels. Potential risks associated with CT radiation in this application could be explored in future studies.

The surface-based approach may be of value in combination with 3D photographing, which does not produce ionizing radiation, and is already used in several contemporary studies^17–19^. The presented approach for selecting the auricle surface from a virtual head may be applicable to 3D photographing as well. Although 3D photographing is hampered by shading artifacts^30^ this may be of lesser importance since the presented surface-based approach only uses the lateral auricle surface, where these effects hardly occur. This enables using the approach for research, using CT images, but also in a clinical setting, using 3D photographing, while providing a larger set of auricle evaluation parameters compared to conventional manual approaches, and with a high precision of measurement. This may be beneficial to refine surgical planning where symmetry and aesthetic outcome is paramount, or in developmental studies, allowing for detailed analyses of craniofacial growth patterns or the assessment of congenital anomalies.

The ANOVA tests show inconsistency among the majority of the mean values of the auricle parameters for the different methods. This variance in difference is largely determined by the fundamentally different ways of measuring auricular parameters. Therefore, researchers and clinicians should be aware of these differences when comparing data obtained through the automatic auricle surface measure based on CT scans with the usual manual caliper measurements. Since in auricular morphology studies there is no established gold standard for aesthetic parameters, we can conclude that the novel approach is more precise but we cannot determine which of the measurement approaches is more accurate. As our study primarily focuses on variability, we highlight the importance of understanding the differences associated with each measurement technique rather than determining an absolute gold standard. Future research may involve establishment of a relationship between measurements obtained using the novel surface-based approach and manual measurements in order to compensate for these differences.

## Conclusion

In conclusion, our study demonstrates that an automated surface-based approach with CT offers superior precision in measuring auricle parameters compared to traditional manual methods based on caliper and 3D landmarking approaches. This automated method has the potential to significantly improve the precision and reliability of auricle reconstruction and evaluation, ultimately benefiting patients undergoing reconstructive surgery, in particular the clinically relevant group of patients with protruding ears.

## Data Availability

The datasets generated during and analysed during the current study are available from the corresponding author on reasonable request.
